# Supplementary material for: Rare X-linked variants carry predominantly male risk in autism, Tourette syndrome, and ADHD
Source: Nat Commun. 2023 Dec 6;14:8077. doi: 10.1038/s41467-023-43776-0 (PMC10700338; doi:10.1038/s41467-023-43776-0)
Supplement: Supplementary file 1 — Supplementary Information [file 41467_2023_43776_MOESM1_ESM.pdf]

Rare X-Linked Variants Carry Predominantly Male Risk in Autism, Tourette Syndrome, and ADHD

Supplementary Figures

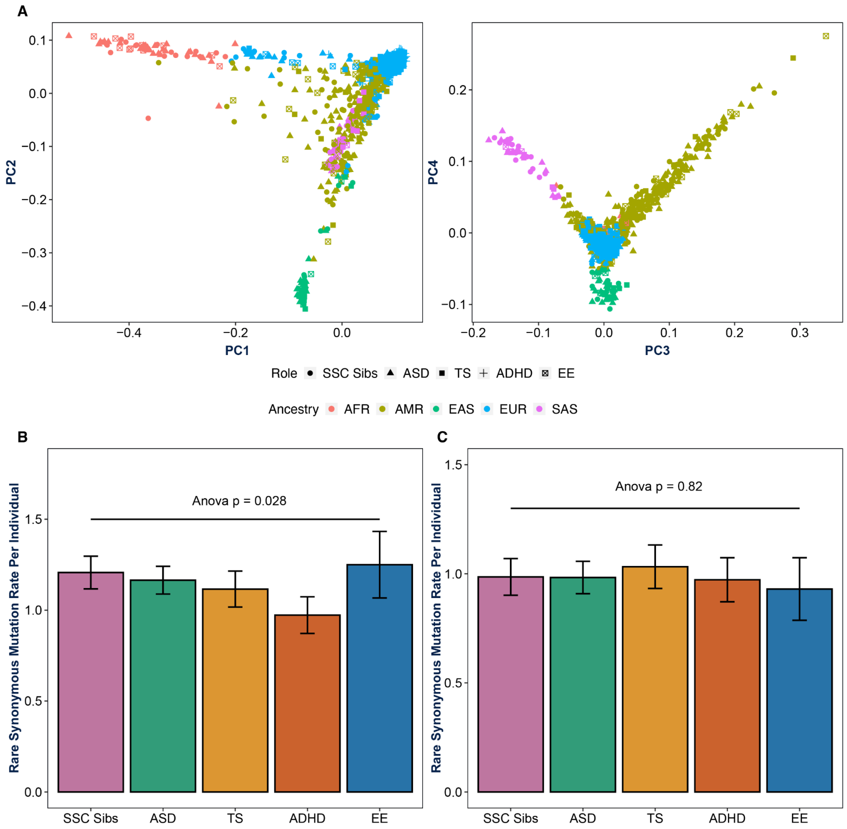

Supplementary Figure 1. Population stratification by dataset and its impact on the rate of rare synonymous variants on Chr X non-pseudoautosomal regions (Chr X non-PAR). (A) We performed principal component analysis for all the male samples in this study, and inferred the population composition with a random forest model, which was trained with data from the 1000 Genomes Project. The majority of our samples were inferred to have European ancestry. AFR, Africans; AMR, Admixed Americans; EAS, East Asians; EUR, Europeans; SAS, South Asians. (B) To ensure that our variant calling pipeline does not lead to batch effects in different datasets, we compared the rare synonymous mutation rate per individual on Chr X non-PAR (730 Simons Simplex Collection siblings or SSC Sibs, 995 ASD probands, 561 Tourette syndrome or TS probands, 329 attention-deficit/hyperactivity disorder or ADHD probands, and 220 epileptic encephalopathies or EE probands). We found that the mutation rates of rare variants from different datasets are significantly different across different datasets, which vary in population stratification (one-way ANOVA, $p = 0.028$). (C) However, when only using individuals from European ancestry, the rare synonymous mutation rates are similar across each dataset (562 SSC siblings or SSC Sibs, 768 Autism spectrum disorders or ASD probands, 494 TS probands, 328 ADHD probands, and 172 EE probands). These results suggest that our variant pipeline does not appear to be introducing biases across datasets and that controlling by synonymous rate should control for differences in ancestry (one-way ANOVA, $p = 0.82$, see also Figure S3). For each bar plot, the height of the bar represents the rare synonymous mutation rate per individual and the black error bars denote the 95% confidence intervals.

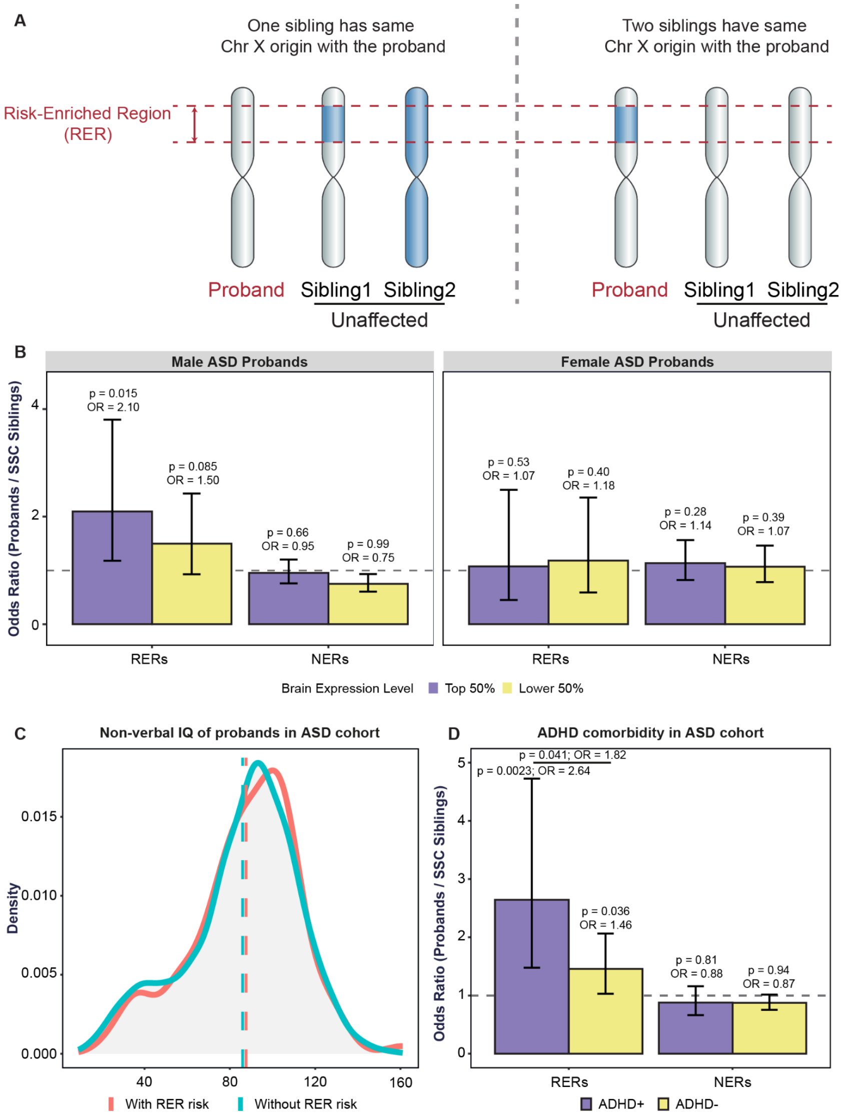

Supplementary Figure 2. The rate of rare damaging variants per individual is elevated in risk-enriched regions (RERs) in probands only. (A) Schema showing how we derived RERs from microarray genotyping data. We included the families with at least three male children and at least one unaffected sibling sharing the same Chr X origin as the proband (N = 48). We then identified regions within the Chr X non-pseudoautosomal regions (non-Par) that consistently segregated with ASD status and termed these RERs. We then termed any regions outside RERs, but within Chr X non-PAR, as non-risk-enriched regions (NERS). (B) Rare transmitted damaging variants tend to be more enriched in RER genes with higher brain expression in males (one-sided Fisher's exact test, 1,014 male probands versus 746 male siblings, 314 females probands versus 811 female siblings). Gene expression levels were estimated from BrainSpan[1,2]. (C) RER risk variants do not appear to impact non-verbal IQ (NVIQ). The distributions of NVIQs from ASD probands carrying RER risk do not appear to differ from those without such risk. The gray shadow highlighted the NVIQ distribution of all ASD probands in SSC. (D) We categorized ASD probands into probands with comorbid ADHD (ADHD+, n = 150) and without comorbid ADHD (ADHD-, n = 830). While both groups show significant enrichment of rare damaging variants in RERs, the mutation rate in the ADHD+ group is significantly higher (one-sided Fisher's exact test). For each bar plot, the height of the bar represents the odds ratio derived from a one-sided Fisher's exact test and the black error bars denote the 95% confidence intervals. P-values are not corrected for multiple comparisons.

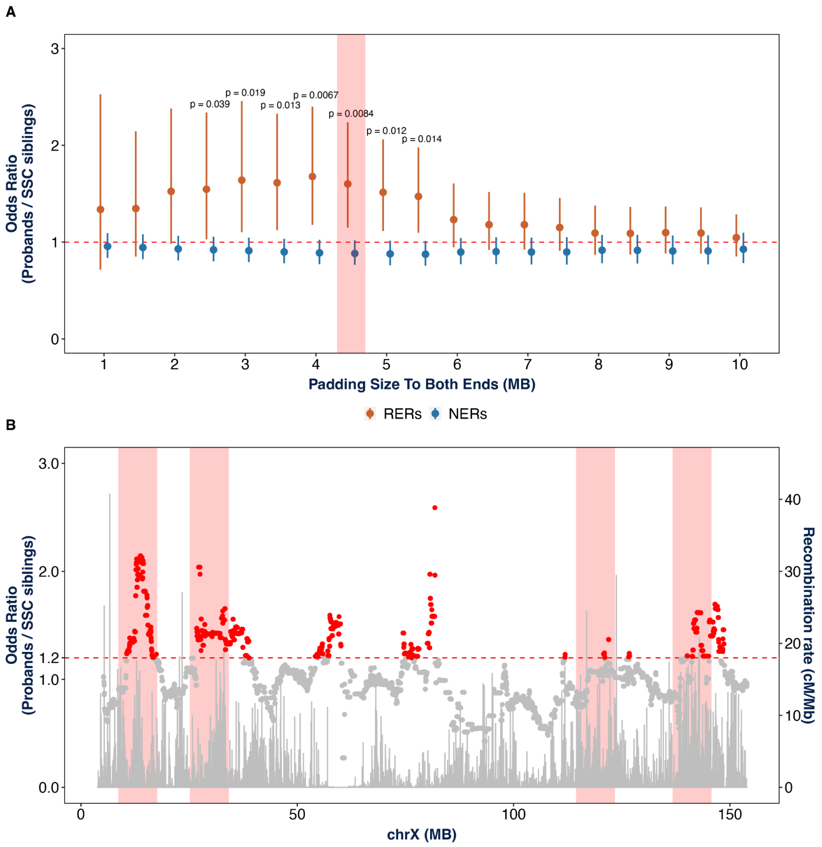

Supplementary Figure 3. Definition of risk-enriched regions (RERs) is solid with varied expansion and with a different method. (A) We conducted the burden study using RERs with various extended region sizes (to both ends of the peak) on the Simons Simplex Collection (SSC) trio dataset (One-sided Fisher's exact test comparing inherited damaging versus inherited synonymous variants in cases versus controls). The enrichment of rare damaging variants is robust as long as the extended region size is not too small where there are not enough variants in RERs for burden analysis. Error bars represent 95% confidence intervals. (B) We utilized an orthogonal sliding window analysis (step = 20kb) with fixed width (width = 5 MB) to determine whether rare damaging variants are over transmitted in Autism spectrum disorders (ASD) cases in specific regions of Chr X. Specifically, we combined variants from SSC and SPARK and compared transmission of rare damaging variants in cases versus controls (Fisher exact test comparing rare maternally-inherited damaging variants versus rare non-inherited variants in cases versus controls). Because each window only had a tiny number of variants, very few windows were statistically significant, although the odds ratio did indicate a general enrichment trend in some windows. Windows with Odds ratio (OR) > 1.2 are highlighted in red (each dot denotes one window). All four RERs (red vertical shadows) overlap multiple windows of interest (OR>1.2). The third RER region only has a few windows, which may be because of the relatively low gene density in that region of Chr X. It is also interesting to note that this analysis identifies two additional regions that may also carry risk. Given the small number of "informative" families (n = 48) used to detect RERs and the low recombination rate in these two regions (due to their proximity to the centromere), we hypothesize that we did not detect these regions in our main analysis due to a lack of power.

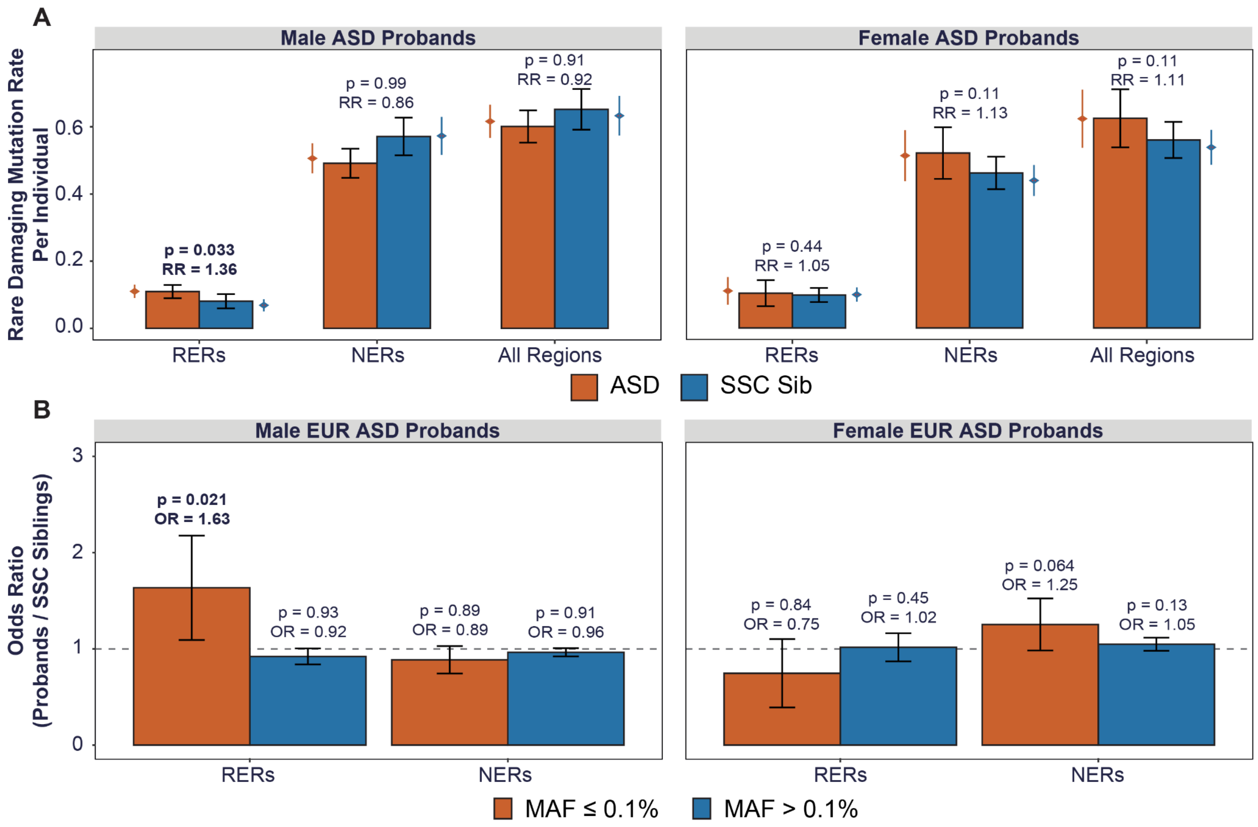

Supplementary Figure 4. Rare transmitted damaging mutation rate is significantly increased in male Autism spectrum disorders (ASD) probands. (A) We compared the rate of rare damaging mutations per individual between ASD probands and Simons Simplex Collection (SSC) siblings, for males (1,014 probands versus 746 siblings) and females (314 probands versus 811 siblings) separately (one-sided t-tests, the height of the bar represents average mutation rate in each group; bar plots; error bars denote 95% confidence intervals). We also show the rate normalized by rare synonymous mutations (red and blue "points" on either side of the bar plots; these are the values compared in the main text). Again, error bars denote 95% confidence intervals. In both cases, the rate of rare damaging mutations is specifically increased in RERs in male probands only, suggesting that normalization by the rate of synonymous variants is not driving our observation of a significant enrichment of rare damaging variants within RERs. (B) Given the population stratification and differences in synonymous variant rates observed in Figure S1, we conducted the main text burden analysis using European samples only (comparing the rate of rare damaging variants normalized by the rate of synonymous variants). Again, we observed significant enrichment for rare damaging variants in RERs from male probands only (one-sided Fisher's exact test, 770 male probands versus 564 male siblings and 238 female probands versus 602 siblings). The height of the bar represents the odds ratio derived from a one-sided Fisher's exact test and the black error bars denote the 95% confidence intervals. The gray horizontal line indicates OR = 1. P-values are not corrected for multiple comparisons.

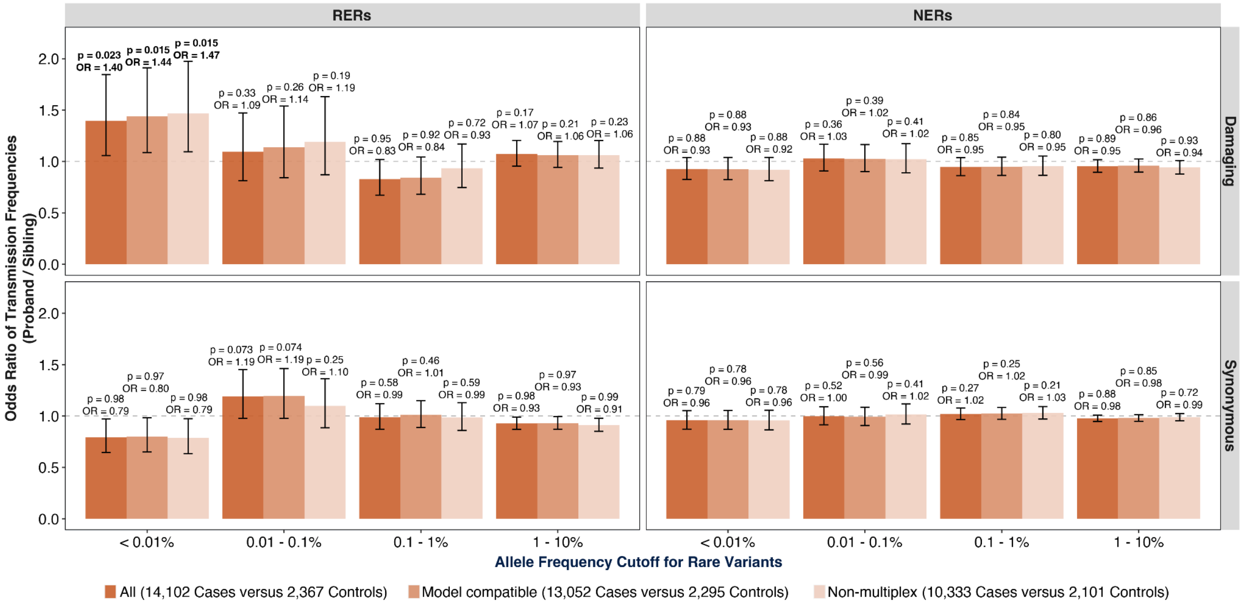

Supplementary Figure 5. Rare damaging variants are over-transmitted to probands in risk-enriched regions only. We filtered rare variants based on different allele frequency thresholds and then conducted burden analyses with Simons Simplex Collection (SSC) and SPARK samples. We repeated this analysis with three different datasets: all male samples, regardless of the family type (All); male samples from SSC (simplex) families, non-multiplex SPARK families, and non-multi-generational multiplex SPARK families with only male affected children (Model compatible); and all male samples but excluding all multiplex families (Non-multiplex). Asterisks indicate significant burden analyses(uncorrected $p < 0.05$, one-sided Fisher's exact test). For each bar, the height of the bar represents the odds ratio derived from a one-sided Fisher's exact test and the black error bars denote the 95% confidence intervals. P-values are not corrected for multiple comparisons.

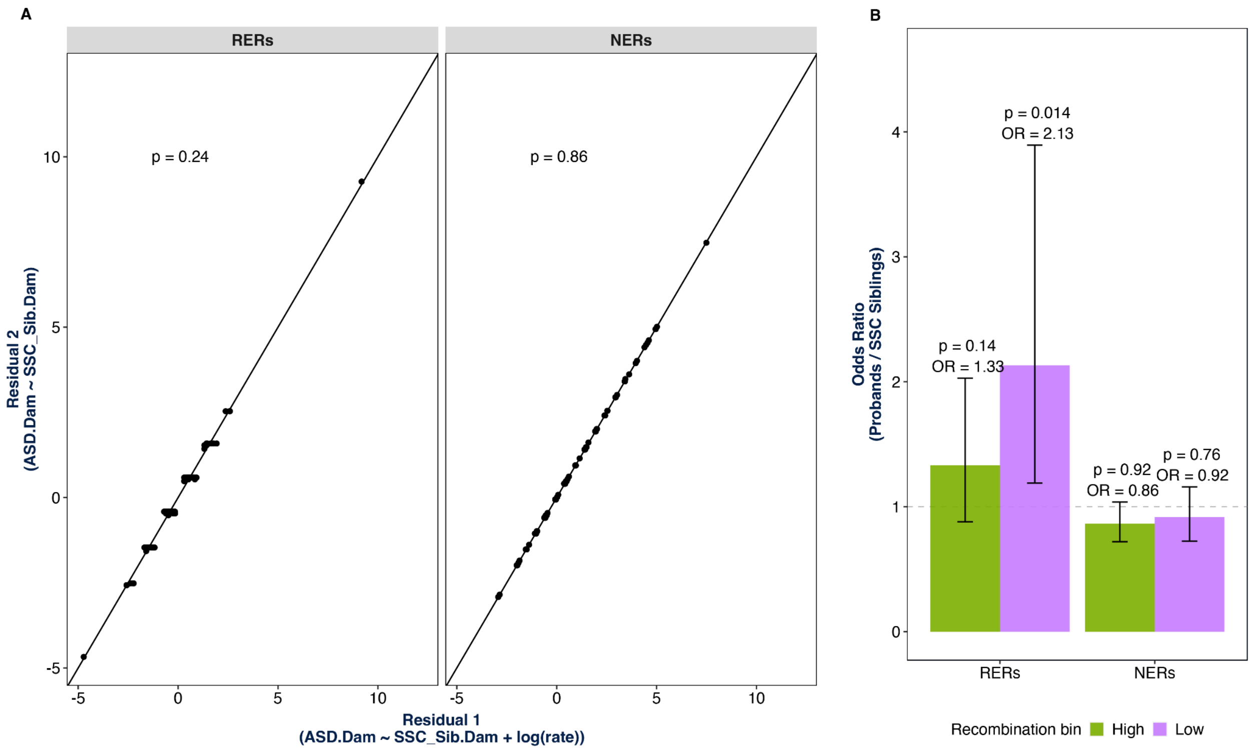

Supplementary Figure 6. Identification of risk-enriched regions (RERs) is not solely dependent on recombination rate. (A) We compared the two regression models with recombination rate as an optional covariate to predict the rare transmitted damaging variants. The recombination rate was transformed to a log scale in order to make its distribution more normal. There is no difference in both RERs and NERs via F-test (p = 0.24 for RERs and p = 0.86 for non-enriched regions or NERs), suggesting that recombination rate is not a significant predictor of the count of rare damaging variants in RERs, NERs. (B) We next split the genes within RERs or NERs into high and low recombination groups and tested the homogeneity of the enrichment of rare damaging variants in ASD probands (1,014 probands versus 746 siblings). To do this, we firstly ranked the genes on Chr X non-pseudoautosomal regions (Chr X non-PAR) based on the averaged recombination rates and grouped the genes into two groups (50-50 split). We did not observe a significant difference in the effects between genes with high and low recombination rate in both RERs and NERs (Breslow-Day test p = 0.24 for RERs, 74 genes in each group; and p = 0.74 for NERs, 328 genes in each group). That being said, the RER genes with a lower recombination appear to trend towards being more enriched, though this is not a conclusive result. For each bar, the height of the bar represents the odds ratio derived from a one-sided Fisher's exact test and the black error bars denote the 95% confidence intervals.

Supplementary Tables

Cohort		# Trios	% AFR	% AMR	% EAS	% EUR	% SAS
SSC Proband	Male	1014	4.34	15.78	2.27	75.94	1.68
SSC Proband	Female	314	4.78	14.97	1.91	75.80	2.55
SSC Sibling	Male	746	4.02	16.22	1.74	75.60	2.41
SSC Sibling	Female	811	4.19	17.26	2.59	74.23	1.73
SPARK Probands	Male	11391	6.48	18.16	2.09	70.47	2.80
SPARK Sibling	Male	1549	2.52	15.43	2.19	76.31	3.55

Supplementary Table 1. Trio samples included in the analysis. SSC, Simons Simplex Collection; AFR, Africans; AMR, Admixed Americans; EAS, East Asians; EUR, Europeans; SAS, South Asians.

Cohort	N	% AFR	% AMR	% EAS	% EUR	% SAS
SSC Proband	995	3.02	15.98	2.21	77.19	1.61
SSC Sibinlg	730	2.60	16.30	1.64	76.99	2.47
TS	561	0.00	8.73	2.14	88.06	1.07
ADHD	329	0.00	0.30	0.00	99.70	0.00
EE	220	5.91	12.27	1.82	78.18	1.82

Supplementary Table 2. Case-control samples included in the analysis (Male only). SSC, Simons Simplex Collection; TS, Tourette syndrome; ADHD, attention-deficit/hyperactivity disorder; EE, epileptic encephalopathies; AFR, Africans; AMR, Admixed Americans; EAS, East Asians; EUR, Europeans; SAS, South Asians.

	Overall in Chr X non-PAR								RERs	
	LGD (AF≤0.25%[1]) (Pro versus Sib)		LGD (AF≤0.1%[2]) (Pro versus Sib)		Mis3 (AF≤0.1%) (Pro versus Sib)		Damaging (AF≤0.1%) (Pro versus Sib)		Damaging (AF≤0.1%) (Pro versus Sib)	
	OR (95% CI)	p-val	OR (95% CI)	p-val	OR (95% CI)	p-val	OR (95% CI)	p-val	OR (95% CI)	p-val
**Male**	1.78 (1.11 - 2.91)	0.019	1.86 (1.08 - 3.30)	0.028	0.94 (0.83 - 1.08)	0.78	0.97 (0.86 - 1.11)	0.65	1.60 (1.15 - 2.24)	0.0084
**Female**	1.19 (0.65 - 2.11)	0.35	1.13 (0.50 - 2.42)	0.46	1.16 (0.96 - 1.39)	0.095	1.16 (0.97 - 1.39)	0.094	1.10 (0.70 - 1.74)	0.40

Supplementary Table 3. Rare transmitted likely gene-disrupting (LGD) variants are enriched in Autism spectrum disorders (ASD) male probands. We conducted a burden analysis for all the rare transmitted variants in Chr X non-pseudoautosomal regions (Chr X non-PAR) or inside the risk-enriched regions (RERs) with a one-sided Fisher's exact test. Mis3: missense variants with PolyPhen2 [HDIV] score ≥ 0.957; Damaging: LGD + Mis3. To reproduce the previous study[3], in addition to the threshold of the rare variants used throughout our study (allele frequency or AF ≤ 0.1%), we included AF ≤ 0.25% in this table to more accurately compare our results to Lim *et al*[3].
[1] AF ≤ 0.25% in our dataset as well as ExAC v0.3; [2] AF ≤ 0.1% in our dataset as well as ExAC v0.3

RER	Start	End
1st	8625243	17625243
2nd	25181023	34181023
3rd	114438272	123438272
4th	136752585	145752585

Supplementary Table 4. Risk-enriched regions (RERs) coordinates in GRCh37. Start and end position of defined RERs.

Chromosome	Role	Number Transmitted	Number Untransmitted	Transmission frequency
Chr X	Case	11431	13297	0.462269
	Ctrl	1743	1980	0.468171
Autosomes (Chr 1-22)	Case	196547	204771	0.489754
	Ctrl	44777	45649	0.495178

Supplementary Table 5. Rare maternally-transmitted synonymous variants on Chr X non-pseudoautosomal regions (Chr X non-PAR) are more undercalled than rare maternally-transmitted synonymous variants on the autosomes. We detected rare maternally-transmitted synonymous variants in SPARK male samples on the autosomes and on Chr X non-PAR using the same criteria described in the Methods. For autosomal variants, the transmitted variants were defined as reference homozygous in father, heterozygous in mother, and heterozygous in child, while the untransmitted variants were defined as reference homozygous in father, heterozygous in mother, and reference homozygous in child. For Chr X non-PAR variants, the transmitted variants were defined as heterozygous in mother, and alternative hemizygous in child, while the untransmitted variants were defined as heterozygous in mother, and reference hemizygous in child.

	Overall in Chr X non-PAR								RERs	
	LGD (AF<=0.25%) (Pro versus Sib)		LGD (Pro versus Sib)		Mis3 (Pro versus Sib)		Damaging (Pro versus Sib)		Damaging (Pro versus Sib)	
	OR (95% CI)	p-val	OR (95% CI)	p-val	OR (95% CI)	p-val	OR (95% CI)	p-val	OR (95% CI)	p-val
**ASD**	1.34 (0.90 - 2.03)	0.12	1.35 (0.85 - 2.18)	0.15	0.92 (0.81 - 1.04)	0.88	0.94 (0.83 - 1.06)	0.82	1.45 (1.05 - 2.00)	0.027
**TS**	1.58 (1.00 - 2.49)	0.047	1.53 (0.90 - 2.59)	0.096	1.01 (0.87 - 1.17)	0.48	1.03 (0.89 - 1.19)	0.38	2.12 (1.46 - 3.08)	0.00032
**ADHD**	1.77 (1.04 - 2.98)	0.041	1.91 (1.04 - 3.46)	0.039	1.14 (0.96 - 1.37)	0.11	1.18 (0.99 - 1.41)	0.062	2.55 (1.60 - 4.08)	0.00032
**EE**	0.60 (0.24 - 1.31)	0.89	0.56 (0.18 - 1.44)	0.91	1.01 (0.83 - 1.23)	0.48	0.99 (0.82 - 1.20)	0.55	1.11 (0.65 - 1.87)	0.41

Supplementary Table 6. Rare LGD variants in male probands. We conducted a burden analysis for all the rare variants in Chr X non-pseudoautosomal regions (Chr X non-PAR) or inside the risk-enriched regions (RERs) with a one-sided Fisher's exact test. LGD: likely gene-disrupting; Mis3: missense variants with PolyPhen2 [HDIV] score ≥ 0.957; Damaging: LGD + Mis3. To reproduce the previous study[3], in addition to the threshold of the rare variants throughout our study (AF ≤ 0.1%), we include AF ≤ 0.25% in this table to more accurately compare our results to Lim *et al*[3].

Supplementary References

1. Hawrylycz, M. J. *et al.* An anatomically comprehensive atlas of the adult human brain transcriptome. *Nature* **489**, 391–399 (2012).
2. Miller, J. A. *et al.* Transcriptional landscape of the prenatal human brain. *Nature* **508**, 199–206 (2014).
3. Lim, E. T. *et al.* Rare complete knockouts in humans: population distribution and significant role in autism spectrum disorders. *Neuron* **77**, 235–242 (2013).
