# Supplementary material for: Rare X-linked variants carry predominantly male risk in autism, Tourette syndrome, and ADHD
Source: Nat Commun. 2023 Dec 6;14:8077. doi: 10.1038/s41467-023-43776-0 (PMC10700338; doi:10.1038/s41467-023-43776-0)
Supplement: Supplementary file 3 — Description of Additional Supplementary Files [file 41467_2023_43776_MOESM3_ESM.docx]

File Name: Supplementary Data 1
Description: Risk gene identification with SSC and SPARK datasets

inRER: whether gene is inside RERs

If ID genes?: whether gene is a known ID gene (Neri et al., 2018)

Trans.Dam.prop: local transmission probability of rare damaging variants in controls

Trans.Syn.prop: local transmission probability of rare synonymous variants in controls

pro.trans.Dam: # of rare transmitted damaging variants in cases

pro.untrans.Dam: # of rare untransmitted damaging variants in cases

pro.trans.Syn: # of rare transmitted synonymous variants in cases

pro.untrans.Syn: # of rare untransmitted synonymous variants in cases

Dam.p.value: one-sided binomial test p value for rare damaging variants using Trans.Dam.prop

Syn.p.value: one-sided binomial test p value for rare damaging variants using Trans.Syn.prop

Dam.p.bonferroni_chrX: Bonferroni's correction for damaging p values by 808 chrX genes

Dam.p.bonferroni_wholegenome: Bonferroni's correction for damaging p values by 19251 genes

Dam.p.FDR_chrX : Benjamini-Hochberg FDR for damaging p values by 808 chrX genes

Dam.p.FDR_wholegenome: Benjamini-Hochberg FDR for damaging p values by 19251 genes

maxExprInAllMaleRegions: maximum median expression percentile in all BrainSpan (Kang et al., 2011) brain regions and time points

maxExprInDevContexts: maximum median expression percentile in ASD related brain regions (Willsey et al., 2013) (all time points) from BrainSpan (Kang et al., 2011)
